# After-action reviews for emergency preparedness and response to infectious disease outbreaks

**DOI:** 10.5365/wpsar.2023.14.1.953

**Published:** 2023-03-22

**Authors:** Ha-Linh Quach, Khanh Cong Nguyen, Florian Vogt

**Affiliations:** a Department of Communicable Diseases Control, National Institute of Hygiene and Epidemiology, Hanoi, Viet Nam.; b National Centre for Epidemiology and Population Health, College of Health and Medicine, Australian National University, Canberra, Australian Capital Territory, Australia.; c The Kirby Institute, University of New South Wales, Sydney, New South Wales, Australia.

Since early 2020, health systems around the world have faced challenges in adequately responding to the coronavirus disease (COVID-19) pandemic. Systems have adapted to the evolving epidemic, and different measures have been implemented at different times in different contexts. Evaluating responses to significant public health events such as outbreaks of infectious diseases is often not prioritized or undertaken due to a lack of resources or time, despite its established importance in improving future preparedness and response measures. (*1*-*3*) Notable examples of evaluations of responses to major infectious disease outbreaks include those for the 2014–2015 (*4*) Ebola virus disease epidemic in the European Union and the 2013 (*5*) H1N1 influenza epidemics in Canada and the United States of America. Evaluations have also been conducted for responses to natural disasters, such as the 2017 wildfires in Portugal (*6*) and Hurricane Katrina in the United States in 2005. (*7*)

In 2015, the World Health Organization (WHO) developed the after-action review (AAR) toolkit as a component of the International Health Regulations (2005; IHR). (*8*-*11*) AARs aim to assess the what, how and why of a response to a significant public health event, to identify the best practices and challenges encountered during the response, and to propose mid- and long-term actions for improvement. The WHO AAR methods were developed to evaluate responses generally to any type of public health event. (*11*) AARs consist of nine pillars for which best practices, challenges and lessons learned are to be identified: (i) country-level coordination and monitoring, (ii) risk communication, (iii) surveillance,  (iv) points of entry, (v) the national laboratory system,  (vi) infection prevention and control, (vii) case management, (viii) operations and logistics and (ix) maintaining health services. (*11*)

Conducting and reporting on an AAR requires three steps: (i) objective observation (i.e. a structured review of response activities); (ii) an analysis of gaps, best practices and contributing factors to the results of the response; and (iii) identification of areas for improvement and proposed follow-up actions. WHO suggests four methods that can be used to conduct an AAR: (i) debriefings,  (ii) working groups, (iii) interviews with key informants and (iv) mixed-methods studies. Depending on the context, AARs can be conducted in different formats and cover different areas of the response. WHO also suggests that the findings of evaluations are compared against the IHR (2005) core capacities. (*11*) Final results should be summarized in a qualitative format, and evaluations by participants contributing to it are encouraged.

It is unclear to what extent WHO’s AAR methods are being used to assess public health responses to events involving emerging infectious diseases and, in particular, how closely such evaluations follow WHO guidance. We undertook a rapid review of the global literature with the objective of understanding how the WHO AAR methods are being used to assess public health responses to infectious disease events.

We searched PubMed using different combinations of keywords such as “after action review,” “infectious disease,” “World Health Organization,” “epidemic,” “outbreak” and “emergency” (**Table 1**). We also searched the WHO Strategic Partnership for Health Security and Emergency Preparedness’ After Action Review database, (*12*) WHO’s main public repository for AARs. We included all articles and reports in English published or uploaded from January 2015 to December 2021 that described using WHO AAR methods to evaluate responses to infectious disease outbreaks. Reports or publications were excluded if they were incomplete, did not use the WHO AAR toolkit, were not published in English or did not evaluate infectious disease events. Results were merged, duplicates removed and the remaining reports screened against the inclusion and exclusion criteria, and reasons for exclusion were documented.

**Table 1:** Search terms used and number of records retrieved from PubMed for study of after-action reviews that use WHO criteria, 2015–2021

Search	Fields searched	Query (filter: English)	No. of records
1	All	after action review	49
2	All	infectious disease	677 493
3	All	epidemic OR outbreak OR emergency	549 053
4	All	World Health Organization	98 489
5	All	infectious disease OR epidemic OR outbreak OR emergency (searches 2 and 3 combined)	1 176 137
6	All	after action review AND infectious disease OR epidemic OR outbreak OR emergency (searches 1 and 5 combined)	20
7	All	after action review AND World Health Organization (searches 1 and 4 combined)	4
8	All	after action review AND infectious disease OR epidemic OR outbreak OR emergency OR after action review AND World Health Organization (searches 6 and 7 combined)	22
9	Date of publication	(2015[Date – Publication]: 2021[Date – Publication])	8 222 679
10	All	after action review AND infectious disease OR epidemic OR outbreak OR emergency OR after action review AND World Health Organization AND (2015[Date – Publication]: 2021[Date – Publication]) (searches 8 and 9 combined)	16

**Fig. 1:**
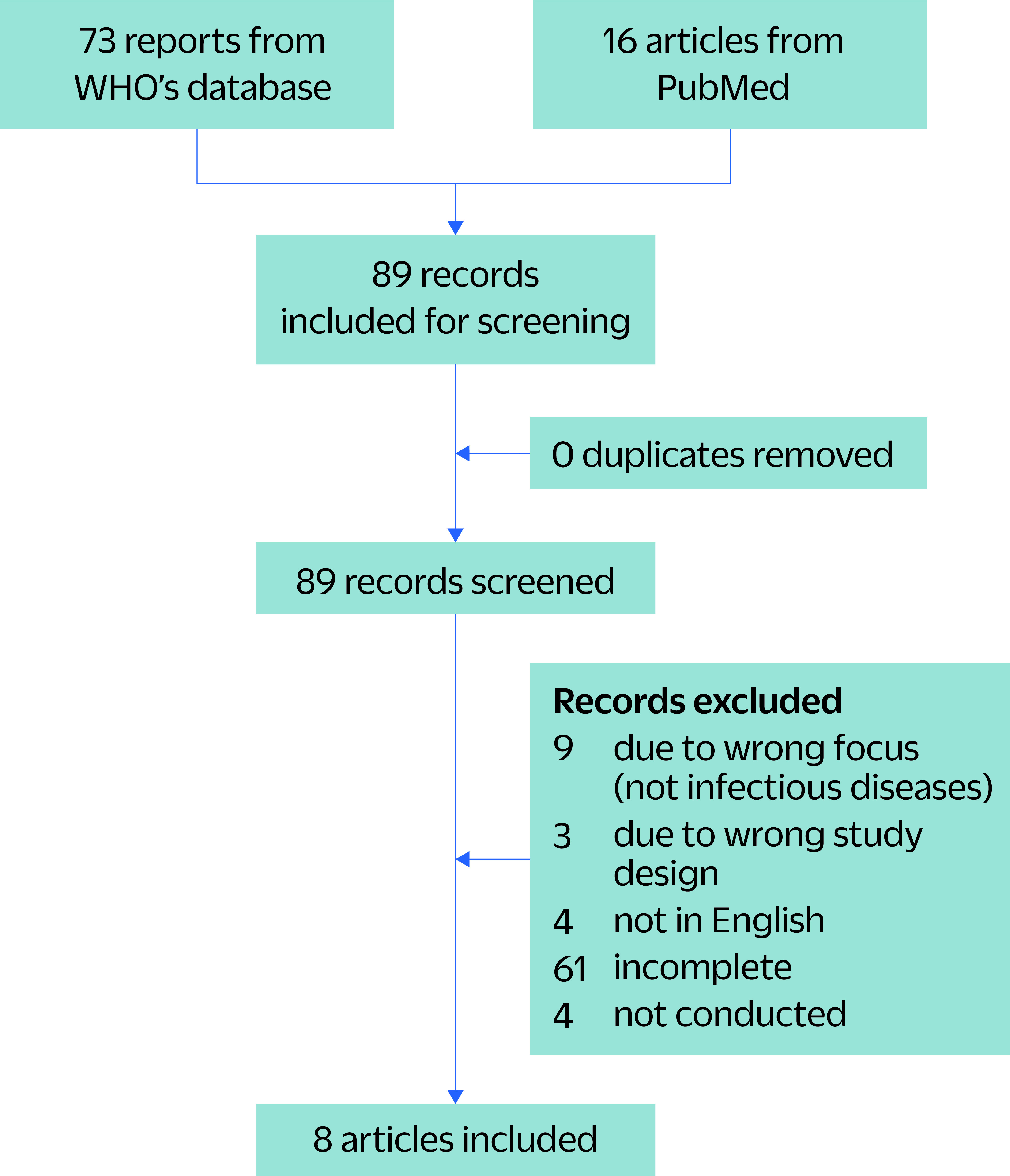
Flow chart of studies and reports retrieved from PubMed for assessment of after-action reviews that use WHO criteria, 2015–2021

For the included reports, we extracted the key characteristics of the AAR method for use in a descriptive analysis. We also assessed how closely the included AARs followed the WHO AAR methods and how effective the methods were in assessing the response. The following data were extracted from each record: general information, including authors and year of publication; setting; scope of evaluation (national, regional or agency level); the event being evaluated; and the year of the event. The reports were then compared against WHO’s AAR guideline (**Table 2**). After screening 86 records,  8 were included in the analysis, 4 from the WHO AAR database and 4 peer-reviewed articles retrieved from PubMed (**Fig. 1**, **Table 3**).

[**insert** Figure 1]

**Table 2:** Data extracted from reports of after-action reviews that use WHO criteria, 2015–2021

Data extracted	Variable
Format	WHO guideline: debriefing, working group, key-informant interviews or mixed-methods study Other
Pillar of evaluation	WHO guideline: (i) country-level coordination and monitoring; (ii) risk communication; (iii) surveillance; (iv) points of entry; (v) the national laboratory system; (vi) infection prevention and control; (vii) case management; (viii) operations and logistics; and (ix) maintaining health services Other
Phases of evaluation	WHO guideline: design, preparation and implementation Other
Comparison with International Health Regulations (2005) core capacities	WHO guideline: Yes No
Final evaluation by participants	WHO guideline: Yes No
Reporting format	WHO guideline: qualitative format with three-part structure: (i) objective observation (i.e. a structured review of response activities); (ii) analysis of gaps, best practices and contributing factors to the results of the response; (iii) identification of areas for improvement and proposals for follow-up actions Other
Follow-up plan for improvement	WHO guideline: Yes No

**Table 3:** Summary of articles and reports included in the study of after-action reviews that use WHO criteria, 2015–2021

Author (year)	Publication type	Setting	Scope of evaluation	Event under evaluation (year)	Evaluation approaches	Areas being evaluated	Application of WHO AAR methodology
Mase et al. (2017) (*13*)	Peer-reviewed journal article	Ohio, USA	Public health departments	H1N1 influenza mass vaccination campaign (2017)	Document review Debriefings Questionaire survey	(1) Mass vaccination (2) Volunteer management (3) Community mitigation (4) Interoperable communica-tions (5) Risk communication (6) Epidemiological surveillance and investigation	£ Followed AAR structure ý Followed AAR pillars for evaluation (focus on vaccination) ý Followed AAR approaches (in combination) £ Comparison with IHR (2005) ý Followed AAR qualitative reporting format £ Final evaluation from participants
Tapo et al. (2021) (*14*)	Peer-reviewed journal article	Vanuatu	International health centre	COVID-19 epidemic (2020)	Document review Debriefing	(1) Coordination and staffing (2) Pre-arrival preparations (3) Pre-departure preparations (point of origin) (4) Upon arrival at the airport in Vanuatu (5) Check in to quarantine facilities (6) During quarantine (7) Quarantine discharge	£ Followed AAR structure ý Followed AAR pillars for evaluation (focus on point of entry) ý Followed AAR approaches (in combination) £ Comparison with IHR (2005) ý Followed AAR qualitative reporting format £ Final evaluation from participants
Boland et al. (2017) (*15*)	Peer-reviewed journal article	Sierra Leone	District health sys-tem, Port Loko district and Kambia district	Ebola virus disease outbreak (2014–2017)	Document review Debriefing Questionnaire survey	(1) Environment and infrastructure (2) Sociocultural aspects (3) Political and organizational aspects	£ Followed AAR structure £ Followed AAR pillars for evaluation ý Followed AAR approaches (in combination) £ Comparison with IHR (2005) ý Followed AAR qualitative reporting format (in combination with quantitative report) £ Final evaluation from participants
Nigeria Centre for Disease Control and WHO (2017)**16**	Non-peer- reviewed report	Nigeria	National public health system	Lassa fever outbreak (2016–2017)	Working groups	(1) Coordination (2) Epidemiological surveillance (3) Case management and infection prevention and control (4) National laboratory system (5) Logistics (6) Risk communication	ý Followed AAR structure ý Followed AAR pillars for evaluation ý Followed AAR approaches £ Comparison with IHR (2005) ý Followed AAR qualitative reporting format £ Final evaluation from participants
Nigeria Centre for Disease Control and WHO (2018)**17**	Non-peer- reviewed report	Nigeria	National public health system	Lassa fever outbreak (2018)	Working groups	(1) Coordination and logistics (2) Case management, safe burial, and infection prevention and control (3) Risk communication and social mobilization (4) National laboratory system (5) Epidemiological surveillance	ý Followed AAR structure ý Followed AAR pillars for evaluation ý Followed AAR approaches £ Comparison with IHR (2005) ý Followed AAR qualitative reporting format ý Final evaluation from participants
Nigeria Centre for Disease Control and WHO (2018)**18**	Non-peer- reviewed report	Nigeria	National public health system	National cerebrospinal meningitis outbreak (2017–2018)	Working groups	(1) Coordination (2) Epidemiological surveillance (3) Case management (4) Risk com-munication and social mobilization (5) National laboratory system (6) Logistics for vaccination	ý Followed AAR structure ý Followed AAR pillars for evaluation ý Followed AAR approaches £ Comparison with IHR (2005) ý Followed AAR qualitative reporting format ý Final evaluation from participants
Nigeria Centre for Disease Control and WHO (2018)**19**	Non-peer- reviewed report	Nigeria	Public health system, Maiduguri Borno state	Cholera outbreak in camp for displaced people (2017)	Working groups	(1) Coordination and logistics (2) Epidemiolo-gical surveillance and the national laboratory system (3) Case management, and infection prevention and control (4) Risk com-munication and community engagement (5) Water, sanitation and hygiene (6) Oral cholera vaccination	ý Followed AAR structure ý Followed AAR pillars for evaluation ý Followed AAR approaches £ Comparison with IHR (2005) ý Followed AAR qualitative reporting format ý Final evaluation from participants
Sorbello et al. (2021)**20**	Peer-reviewed journal article	Italy	Hospital of San Raffaele Scientific Institute, Milan	COVID-19 epidemic (2020)	Key-informant interviews	(1) Staff management (2) Logistics and supplies (3) COVID-19 diagnosis and clinical management (4) Risk communication	ý Followed AAR structure ý Followed AAR pillars for evaluation (modified to quantitative ranking of effectiveness) ý Followed AAR approaches £ Comparison with IHR (2005) £ Followed AAR qualitative reporting format ý Final evaluation from participants

AAR: after-action review; IHR: International Health Regulations; WHO: World Health Organization.

Three AARs (*13*-*15*) used WHO-recommended methods in combination with other evaluation tools, such as document reviews or surveys in addition to quantitative assessments. The remaining five (*16*-*20*) strictly followed WHO’s nine evaluation pillars and three steps, and were conducted as conferences that brought together all stakeholders. Four of the eight reports used working groups, (*16*-*19*) three used debriefings (*13*-*15*) and one used key-informant interviews, (*20*) following WHO’s AAR ready-to-use toolkits.

Public health systems were a common focus of evaluations, appearing in seven AARs, (*13*-*19*) while another AAR focused on a hospital setting. (*20*) Five AARs were conducted at the local level in response to outbreaks (*13*-*15*, *19*, *20*) and three at the national level. (*16*-*18*)

Three AARs included participants’ evaluations of and feedback on the AAR method. (*17*-*19*) Although the overall assessment of the suitability of AARs to connect stakeholders, provide a platform for ideas and to pool experiences was positive, as evidenced by responses from more than 80% of participants in each of these three studies, only half of the participants agreed that AARs actually achieved their objectives. (*17*-*19*) In terms of strengthening interdisciplinary collaboration and coordination, less than 20% of participants in these studies rated this as being accomplished by the AAR. (*17*-*19*) Additionally, the importance of allowing AAR methods to be adjusted to best fit their purposes (e.g. for smaller-level analyses, such as within a unit, region or single institution) is highlighted by the fact that three of the eight reports did not strictly follow WHO’s AAR structure. (*13*-*15*) The importance of making modifications to conduct a more focused system evaluation was also flagged by Sorbello et al. (*20*) as a way to improve follow-up actions within local contexts and to enhance multidisciplinary cooperation. Despite being recommended by WHO, none of the AARs used the IHR (2005) core capacities as a comparator.

Only a limited number of AARs have been published in the scientific and grey literature. As of August 2022, the global public repository for AARs at WHO (*12*) listed 81 entries since 2016. However, 66 (88%) of the 75 entries categorized as having been conducted were incomplete, of which 41 were older than 2 years and hence are unlikely to ever be finalized. Furthermore, many entries had only minimal information about the setting and category of emergency, and were without much content about the AAR itself. It was also often difficult to establish whether an AAR had actually been conducted and completed successfully. The problem of identifying and accessing information about AARs has also been recognized in a recent review from Australia. (*21*)

From the completed reports, it is evident that WHO’s methods were not always strictly followed, but they were often used in combination with other methods for qualitative and quantitative assessment. While the qualitative element of WHO’s AAR toolkit seems to have been easier to follow in conference settings with all relevant stakeholders present, several AARs required methodological modifications, using, for example, surveys or document reviews, and also incorporated quantitative methods, depending on the local context. One of WHO’s main recommendations for conducting AARs is to compare the outcomes of the response with the IHR (2005) core capacities – a country-level assessment – yet this comparison was not done in any of the studies included in our analysis.

WHO’s AAR methodology is relatively broad and geared towards whole-of-system evaluations. The nine AAR evaluation pillars and the accompanying toolkit are also rather general. As a result, AARs were more frequently used for evaluations of district- and national-level systems rather than for specific systems (e.g. surveillance systems, national laboratories or point-of-entry screening). However, assessments should be conducted for all levels and aspects of health systems to ensure a comprehensive response; therefore, AAR toolkits should be flexible enough to be adapted to different jurisdictions and scopes of assessment to accommodate diverse evaluation needs. Thus, modifications to WHO’s AAR guidance are important to ensure that relevant information can be gathered from a wider range of sources and a more diverse group of stakeholders to fully consider local contexts and different scopes of evaluation. Furthermore, understanding IHR (2005) core capacities could offer important lessons for conducting AARs. However, comparison against IHR (2005) core capacities is rarely done as part of AARs despite being encouraged by WHO. We also found that several AARs in the WHO repository were implemented without assessments from participants and stakeholders.

WHO issued a modified version of its methods for AARs at the beginning of the COVID-19 pandemic, known as intra-action reviews (IARs), to meet the need to rapidly assess health systems’ performance during the ongoing pandemic. As of August 2022, there were 144 IARs listed in the WHO database; (*22*) 129 of them (89.6%) were categorized as conducted, but only 19 of these (14.7%) were accompanied by a completed report, suggesting there are issues in finalization and publication similar to those for AARs. IARs include four additional pillars that are relevant to the ongoing COVID-19 pandemic: (i) COVID-19 vaccination, (ii) vulnerable and marginalized populations, (iii) national legislation and financing, and (iv) public health and social measures. (*23*) However, only two IARs categorized as conducted in the database included information about COVID-19 vaccination, and none of the IARs provided information about the other three pillars. Therefore, it is unclear whether IARs have contributed to improving evaluations of health system responses. The IAR adaptation of the AAR remains relatively broad and geared towards national-level responses. Similar to AARs, we believe that IARs would greatly benefit from regular evaluation of the methodology itself to better guide and prepare countries and health-care systems for future, protracted health emergencies beyond the COVID-19 pandemic. (*23*)

We acknowledge several limitations to our work. First, the small number of included records did not allow for strong conclusions. Second, there were many AARs listed in the WHO repository that did not have a completed report, which again led to only a small number of records being included in our study and possible publication bias in our assessment. Third, the range of countries with completed AARs was limited and quite focused on WHO’s African Region, which restricts the generalizability of our findings. Fourth, less than half of the included studies reported on participants’ evaluations of AARs, which hindered our ability to obtain sufficient information about their reflections on the suitability of the AAR method to achieve its objectives. All of these constraints could be resolved through more stringent reporting requirements for AARs. Unfortunately, there is no formal requirement to report on and publish AARs upon completion or to finalize reports in a timely manner. Especially during the COVID-19 pandemic, as more and more health systems need up-to-date data on effective and ineffective measures for addressing the pandemic, it is important to disseminate these evaluations widely and rapidly to ensure that incremental and strategic improvements are made to health-care systems worldwide. However, it seems that COVD-19-specific IARs suffer from the same issues as AARs in terms of insufficient conclusions and lack of publication of reviews.

It is crucial to evaluate public health systems regularly during a prolonged and evolving event such as the COVID-19 pandemic. Selecting appropriate methods for these evaluations is important to their successful implementation and to ultimately improve and adapt responses to the pandemic. Considering the variability of the COVID-19 pandemic and countries’ public health capacities, a global methodology such as WHO’s AAR toolkit needs to be sufficiently adaptable to local contexts and priorities, and also able to gain the most value from stakeholders’ practical experiences during the response. The COVID-19-specific IAR adaptation of the AAR is a laudable example of this type of approach, and future pandemics might require similar adaptations. Furthermore, more subnational reviews, which have been proposed in the latest version of the IAR, are needed to enable better operational analysis of public health responses in specific high-priority areas. Importantly, the reporting and publication of completed AARs should be strengthened to allow public health responders and researchers from other countries and settings to benefit from the knowledge generated and lessons learned to strengthen the capacities of health-care systems to respond to future health emergencies. (*23*)
